# Industrial Potential of Formaldehyde Gas Sensor Based on PdPt Bimetallic Loaded SnO_2_ Nanoparticles

**DOI:** 10.3390/s25051627

**Published:** 2025-03-06

**Authors:** Bing Shen, Tongwei Yuan, Wenshuang Zhang, Xian Tan, Yang Chen, Jiaqiang Xu

**Affiliations:** 1 NEST Lab, College of Science, Shanghai University, Shanghai 200444, China; bingshen@shu.edu.cn (B.S.); tongwei@shu.edu.cn (T.Y.); zhangwenshuang@shu.edu.cn (W.Z.); 2 Key Laboratory of Organic Compound Pollution Control Engineering (MOE), School of Environmental and Chemical Engineering, Shanghai University, Shanghai 200444, China; tanxian2023@shu.edu.cn

**Keywords:** formaldehyde detection, gas sensor, SnO_2_, PdPt noble metals

## Abstract

**Highlights:**

- Gas-sensing material with high stability for industrial potential.
- Portable gas sensor device from laboratory to practical use.

**What are the main findings?**

- SnO_2_ decorated with PdPt bimetallic nanoparticles can be produced facilely.
- The portable device based on the PdPt-SnO2 sensor can detect HCHO in real space.

**What is the implication of the main finding?**

- The facile synthesis method could be adopted by industrial producing.
- Gas sensor research could proceed from laboratory to industrial use.

**Abstract:**

SnO_2_-based semiconductor gas-sensing materials are regarded as some of the most crucial sensing materials, owing to their extremely high electron mobility, high sensitivity, and excellent stability. To bridge the gap between laboratory-scale SnO_2_ and its industrial applications, low-cost and high-efficiency requirements must be met. This implies the need for simple synthesis techniques, reduced energy consumption, and satisfactory gas-sensing performances. In this study, we utilized a surfactant-free simple method to modify SnO_2_ nanoparticles with PdPt noble metals, ensuring the stable state of the material. Under the synergistic catalytic effect of Pd and Pt, the composite material (1.0 wt%-PdPt-SnO_2_) significantly enhanced its response to HCHO. This modification decreased the optimal working temperature to as low as 180 °C to achieve a response value (*R*_a_/*R*_g_ = 8.2) and showcased lower operating temperatures, higher sensitivity, and better selectivity to detect 10 ppm of HCHO when compared with pristine SnO_2_ or single noble metal-decorated SnO_2_ sensors. Stability tests verified that the gas sensor signals based on PdPt-SnO_2_ nanoparticles exhibit good reliability. Furthermore, a portable HCHO detector was designed for practical applications, such as in newly purchased cushions, indicating its potential for industrialization beyond the laboratory.

## 1. Introduction

With the progress of society, the living standards of humans have been steadily improving. Nevertheless, science and technology are like a double-edged sword. While people relish the convenience they bring, they also give rise to a series of environmental problems. Among these, gas pollution stands out as a grave concern. It not only damages the ozone layer and exacerbates the greenhouse effect but can also trigger negative consequences such as acid rain. Moreover, it exerts a significant impact on human health. Formaldehyde can impact human health through multiple pathways and is regarded as one of the most severely harmful gases. Once inhaled into the human body, it exerts toxic effects on the cardiovascular system, the endocrine system, and other aspects [1,2,3]. Therefore, in addition to enhancing the management system for toxic and harmful gas emissions, the development of advanced gas detection and alarm devices is both urgent and highly significant for the real-time monitoring of hazardous substances.

Currently, there exist calibration methods for air pollutants, such as infrared optical analysis and gas chromatography detection. These testing methods possess extremely high accuracy and low detection limits. However, owing to their complex operational procedures, they can only be operated by professionals at very few specific sites [4,5]. After years of research efforts, gas sensors featuring simple preparation methods and low costs have entered the market. Among these, metal oxide semiconductor (MOS) gas sensors stand out, with their remarkable advantages of miniaturization, ease of integration, and a wide range of applications [6,7,8]. SnO_2_, a typical wide-bandgap (3.6 eV) semiconductor material, exhibits high electron mobility. Consequently, it is frequently utilized in MOS gas sensors for detecting a variety of combustible gases, industrial exhaust gases, and environmentally harmful gases [9,10]. Nevertheless, pristine SnO_2_ fails to meet the requirements in specific environments. Surface modification with noble metals, recognized as one of the most effective ways to enhance the performance of gas-sensitive materials, has been demonstrated to facilitate the interaction between sensitive materials and gases [11,12]. Especially, the modification with dual noble metals assumes a crucial role in this domain. Due to their unique synergistic effect, Pd and Pt bimetallic noble metals are often jointly employed to modify sensing materials, thereby enhancing their performance. Xu group utilized PdAu-decorated 3D SnO_2_ for 100 ppm HCHO detection with a response value of 125 [13], and Li et al. synthesized PdPt onto SnO_2_ nanosheets for 50 ppm CO and 500 ppm CH_4_ dual-selectivity detection at different temperatures [14]. Moreover, a large proportion of scientific research work is limited to laboratories, but industrial informatization requires the research and development of handheld or wearable devices [15,16,17,18].

In this study, we employed an extremely simplified hydrothermal method to synthesize 1.0 wt%-PdPt-SnO_2_. This approach significantly enhanced the sensing stability towards formaldehyde and substantially reduced the working temperature. Our work offers a simple yet effective method for the synthesis of sensing materials and holds the potential for more stable long-term detection of formaldehyde.

## 2. Materials and Methods

### 2.1. Synthesis of PdPt-SnO_2_

Analytical grade tin tetrachloride pentahydrate (SnCl_4_·5H_2_O), glucose (C_6_H_12_O_6_), palladium dichloride (PdCl_2_), and chloroplatinic acid (H_2_PtCl_6_·xH_2_O) were purchased from Sigma-Aldrich Merck Limited, Shanghai, China. Ethylene glycol (C_2_H_6_O_2_) was obtained from Shanghai Sinopharm Chemical Reagent Co., Ltd., Shanghai, China. Methane (pure CH_4_), carbon monoxide (pure CO), ammonia (NH_3_/N_2_, 300 ppm), nitrogen monoxide (NO/N_2_, 100 ppm), hydrogen sulfide (H_2_S/N_2_, 100 ppm), and mixed formaldehyde/nitrogen dry gas (HCHO/N_2_, 500 ppm) were purchased from Dalian Special Gases Co., Ltd., Dalian, China, and the error tolerance of the gas used was around ±2% mol. Ultra-pure water (18.2 MΩ) from a Milli-Q System (Millipore, Boston, USA) was used in the experiments.

In a complete synthesis process, 70 mL of ultra-pure water was poured into a beaker. Subsequently, 0.65 g of SnCl_4_·5H_2_O, 1.9817 g of glucose, and 0.0032 g of PdCl_2_ were added. Using a pipette, 2.3 mL of H_2_PtCl_6_ solution was then introduced, and the mixture was stirred at room temperature for 3 h. Next, the solution was transferred into a 100 mL Teflon-lined stainless-steel autoclave. The autoclave was heated at 180 °C for 16 h. After that, the resulting material was centrifuged and alternately washed with ultra-pure water and ethanol. It was then placed in a vacuum drying oven at 70 °C for 12 h. Finally, the material was transferred to a tube furnace. It was heated at a rate of 2 °C/min to 500 °C and held at this temperature for 180 min. After naturally cooling down to room temperature, the powder material was collected and labeled as 1.0 wt%-PdPt-SnO_2_. Furthermore, the above experimental steps were repeated with modifications to the amounts of PdCl_2_ and H_2_PtCl_6_ added. Two types of nanomaterials with different noble metal contents were synthesized. For the synthesis of 0.5 wt%-PdPt-SnO_2_, 1.15 mL of H_2_PtCl_6_ solution and 0.0016 g of PdCl_2_ were used. For the synthesis of 1.5 wt%-PdPt-SnO_2_, 3.45 mL of H_2_PtCl_6_ solution and 0.0048 g of PdCl_2_ were employed, respectively.

### 2.2. Characterization

The powder X-ray diffraction pattern (XRD) of the sample was recorded on a Rigaku Miniflex (Tokyo, Japan) 600 at a speed of 0.02°/s. The device operates under 40 kV voltage and 15 mA current conditions, with Cu K_α1_ radiation (λ = 0.15406 nm), with a scanning angle range of 10–80°. Transmission electron microscope (TEM) and high-resolution TEM (HRTEM) were carried out on a JEM-2010F electron microscope, which was operated at 200 kV. Energy dispersive X-ray spectroscopy (EDX) images of the samples were acquired in a JEM 2100F machine at an acceleration voltage of 100 kV. The field emission scanning electron microscope (FESEM) was observed on a JSM-6700F SEM. The inductively coupled plasma optical emission spectrometer (ICP-OES) measurement was performed on an Optima (Shelton, CT, USA) 7300 DV. X-ray photoelectron spectra (XPS) studies were determined by a Thermofisher (Waltham, MA, USA) equipment (WSCALAB), and the C 1s signal at 284.8 eV was used to calibrate the binding energy scale.

### 2.3. Fabrication and Sensing Measurements of Gas Sensors

#### 2.3.1. Manufacturing of Gas-Sensing Devices

**Scheme 1:**
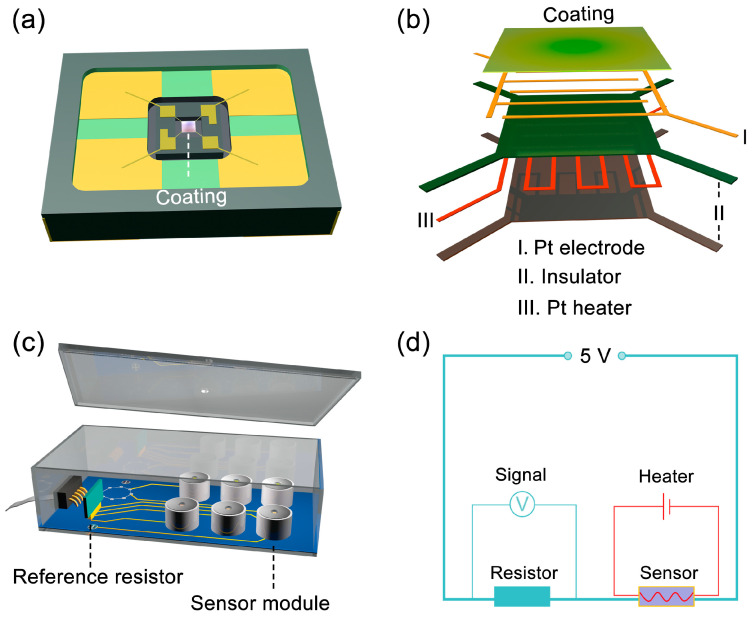
Schematic diagram of (**a**) MEMS gas sensor, (**b**) layer structure of core part, (**c**) gas sensor testing chamber, and (**d**) design circuit.

Once the materials have been collected, carefully measure out an appropriate quantity and transfer it into a mortar. Next, introduce a small volume of ethylene glycol gradually in a drop-by-drop manner. Thoroughly grind the mixture for a duration of 30 min to ensure a homogeneous blend. After the grinding process is complete, employ a syringe to precisely dispense the prepared mixture into the core section of the MEMS sensor. Allow the sensor with the added mixture to remain undisturbed for 2 h to enable proper settling and interaction. Subsequently, place the MEMS sensor in a muffle furnace. Set the furnace to a temperature of 250 °C and conduct the calcination process for 2 h to achieve the desired chemical and physical changes. Scheme 1a is the schematic diagram of the structure of the MEMS (micro-electro-mechanical systems), where the central area is the sensing coating made from our synthesized nanomaterials, assembled with the signal acquisition circuits and heating circuits. Scheme 1b is the schematic diagram of the specific structure of the heating coating, where [I] is the Pt electrode to collect the response signals, [II] are the insulation layers, and [III] is the Pt heating layer. Scheme 1c is the schematic diagram of the testing device’s structure. There is a load resistance card at the left of the testing box to hold the load resistance, and on the right is the module device of the load chip. The coated components are placed into the module to form a closed circuit. Scheme 1d is a schematic diagram of the sensor circuit. The sensor is connected in series with a constant load resistor at a total voltage of 5 V. The heating circuit passes through the sensor and is independent of the testing system. The working temperature of the sensor is changed by adjusting the heating voltage. By measuring the output voltage on the load resistor connected in series with the gas sensitive element, the resistance change in the gas sensitive element is calculated, thereby reflecting the characteristics of the gas sensitive element. The response value of the gas-sensing element is Response = *R*_a_/*R*_g_, where *R*_a_ and *R*_g_ are the resistances of the element in air and in the tested atmosphere, respectively.

#### 2.3.2. Testing of Gas-Sensing Performance

An appropriate quantity of the sensing material was taken, and an adequate amount of pine oil alcohol was added. After thorough grinding, the ground mixture was applied onto the sensing layer of the device. Subsequently, the device was placed into a muffle furnace for sintering. It was annealed at 250 °C for 2 h and then allowed to cool down to room temperature. After that, the devices were connected to an aging table, and the sensors were aged at 250 °C for 5 days prior to conducting any testing.

## 3. Results

### 3.1. Morphology and Material

**Figure 1:**
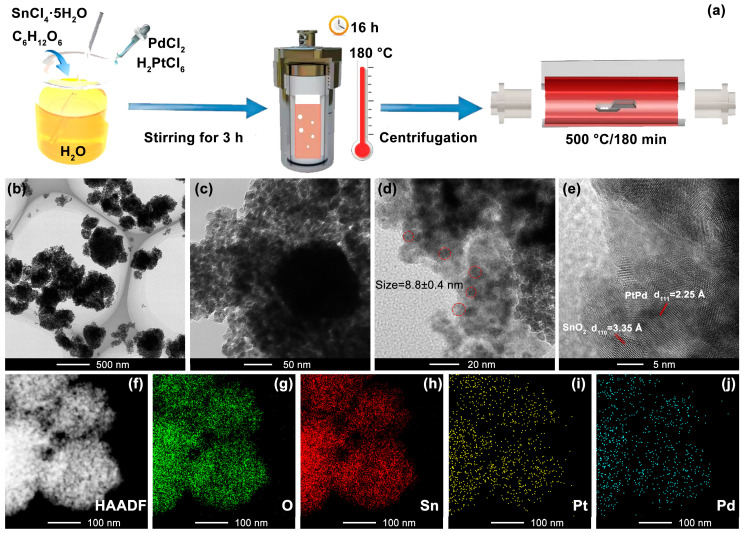
(**a**) The diagrammatical illustration of the facile synthesis method of PdPt-SnO_2_. (**b**–**e**) TEM and HRTEM images for 1.0 wt%-PdPt-SnO_2_. (**f**–**j**) EDX elemental mapping of 1.0 wt%-PdPt-SnO_2_.

Gas sensors commonly utilize nanomaterial coatings because of their high surface activity. However, excessively complex synthesis processes may lead to increased costs and reduced reproducibility. More importantly, special morphologies frequently exhibit thermodynamic instability, which causes significant performance deterioration of the sensors over time [19]. This work adopts a simple one-pot method to synthesize PdPt bimetal-modified SnO_2_ particles without the use of surfactants (Figure 1a). This approach enables the nanomaterials to exist in their most natural thermodynamic state, thereby significantly enhancing long-term thermal stability. SnO_2_ is the metal oxide with the greatest industrial potential, and the PdPt bimetal modification can considerably enhance sensitivity and selectivity. As depicted in Figure 1b–e, the TEM images with different scales confirm that the synthesized 1.0 wt%-PdPt-SnO_2_ material shows a relatively natural nanoparticle morphology, with an average particle size around 8.8 ± 0.4 nm. HRTEM analysis reveals the lattice fringes of SnO_2_ and PdPt bimetals, with d_110_ = 3.35 Å and d_111_ = 2.25 Å, respectively. As depicted in Figure 1f–j, the EDX characterization indicates that the Sn and O elements are intertwined as the substrate material, while the Pd and Pt elements are uniformly distributed as the sensitivity-enhancing materials [20,21].

**Figure 2:**
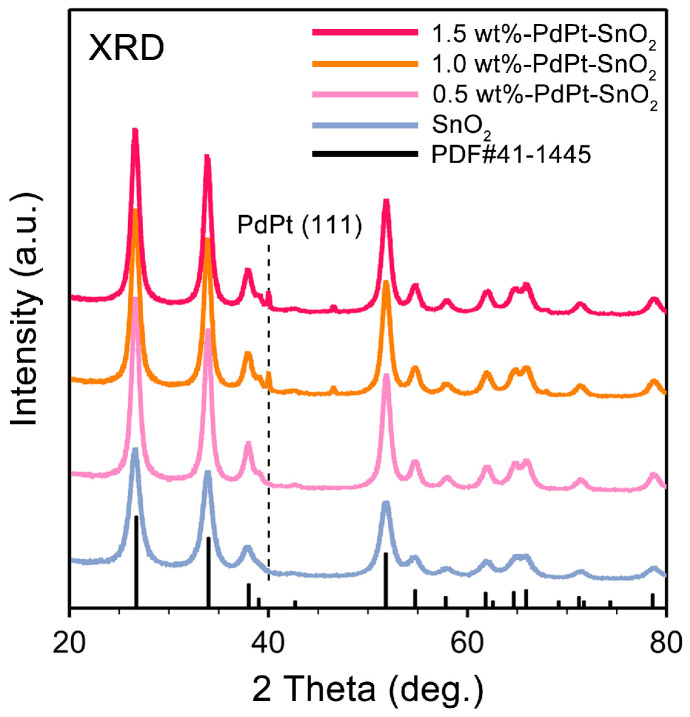
XRD patterns of pristine SnO_2_ nanoparticles and PdPt-decorated SnO_2_ materials.

The XRD patterns of four materials are presented in Figure 2 and Figure S1. All samples show strong diffraction peaks corresponding to SnO_2_, which match the PDF card (#41-1445) clearly, confirming that the synthesized nanomaterial is SnO_2_ with a crystal structure belonging to the spatial group of P42/mnm (136). The Pd or Pt only gives small peaks near to 40°, which match the standard cards Pd (#00-005-0681) and Pt (#00-004-0802), respectively. And the PdPt bimetallic nanoparticles show a peak at 39.9°, belonging to the (111) crystal plane. And the presence of Pd and Pt is clearly definite in the HRTEM images and EDS mapping discussed earlier. When combined with the inductively coupled plasma optical emission spectroscopy (ICP-OES) data, which provide the precise element distribution (Table 1), single noble metal-decorated SnO_2_ materials contain about 1.0 wt% Pd or Pt contents, and the total Pd and Pt loading of bimetal loaded materials is approximately 0.5 wt%, 1.0 wt%, and 1.5 wt%, respectively, with their ratios being nearly 1:1.

**Figure 3:**
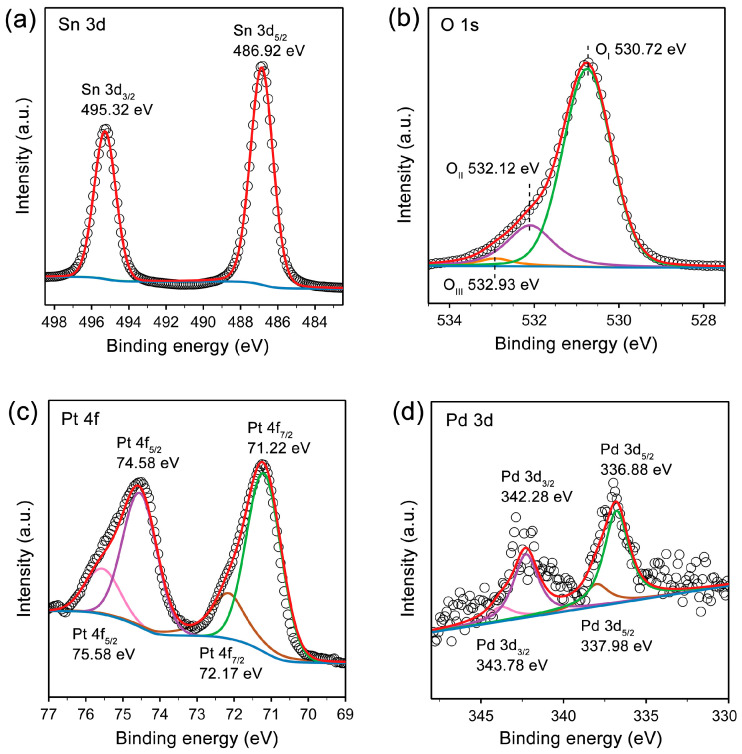
XPS fine-scanning spectra and fitting peaks of 1.0 wt%-PdPt-SnO_2_: (**a**) Sn 3d, (**b**) O 1s, (**c**) Pt 4f, and (**d**) Pd 3d. (Circle dots are the raw data, red lines are the fitting results, blue lines represent backgrounds, and others are the contributions from different signals).

Subsequently, we examined the surface chemical composition of the 1.0 wt% PdPt-SnO_2_ using X-ray photoelectron spectroscopy (XPS). As shown in Figure S2, the primary elements present in the nanomaterial are O, Sn, Pd, and Pt, with each element exhibiting its characteristic peaks and no detectable impurity peaks (except for carbon). The high-resolution Sn 3d spectrum (Figure 3a) reveals two peaks at 495.32 eV and 486.92 eV, corresponding to Sn 3d_3/2_ and Sn 3d_5/2_, respectively. Figure 3b shows the surface states of the oxygen element. O_I_, O_II_, and O_III_ represent three different states of surface oxygen, where O_I_ corresponds to lattice oxygen, O_II_ corresponds to vacancy oxygen, and O_III_ corresponds to adsorbed oxygen. The binding energies of these species are 530.72 eV, 532.12 eV, and 532.92 eV, respectively. Notably, defect and vacancy oxygen are expected to contribute to enhancing the gas-sensing performance of the material [22,23].

Figure 3c shows the Pt 4f spectrum, with characteristic peaks corresponding to Pt 4f_7/2_ and Pt 4f_5/2_ at 71.2 eV and 74.6 eV, respectively. The peaks at 71.2 eV and 74.6 eV are attributed to metallic Pt^0^, while the peaks at 72.2 eV and 75.6 eV correspond to Pt^2+^, confirming the presence of both Pt^2+^ and Pt^0^ in the 1.0 wt%-PdPt-SnO_2_ sample. Similarly, Figure 3d shows the Pd 3d spectrum, with characteristic peaks for Pd 3d_5/2_ and Pd 3d_3/2_ at 336.8 eV and 342.3 eV, respectively. The peaks at 336.88 eV and 342.28 eV correspond to metallic Pd^0^, while the peaks at 337.98 eV and 343.78 eV are attributed to Pd^2+^. Pt and Pd primarily exist in the zero-valent elemental state, with a small amount of oxidation present in the divalent state on the surface, confirming that the material is PdPt-SnO_2_.

### 3.2. Gas-Sensing Performances

**Figure 4:**
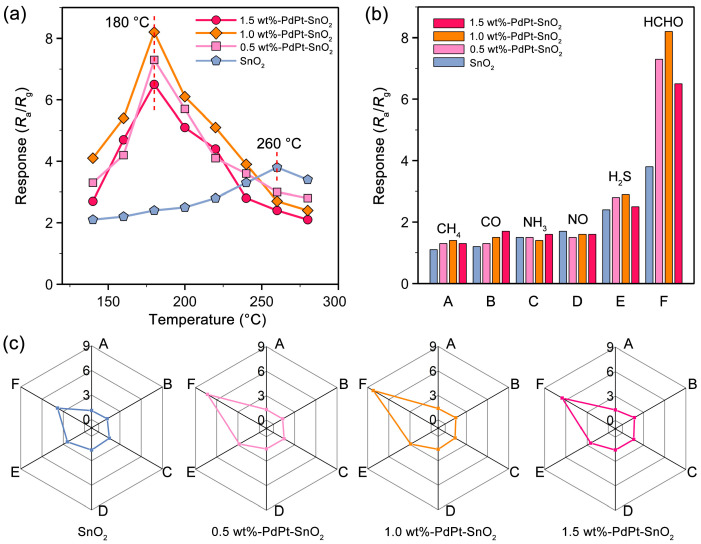
(**a**) Responses at various working temperatures, (**b**) selectivity tests to 10 ppm of different gases, and (**c**) transformed radar charts for the sensors based on SnO_2_ and PdPt-decorated SnO_2_ nanoparticles in response to 10 ppm HCHO.

After preparing the nanomaterials into a slurry, it is coated on the surface of the MEMS sensor and then undergoes annealing treatment to fabricate a gas sensor (Figure S3). For semiconductor gas sensors, the selection of the operating temperature is crucial. At lower temperatures, there may not be enough chemical energy to drive the reaction of semiconductor oxides, resulting in a weak chemical activation effect. On the other hand, high temperatures can cause the adsorbed gas to desorb before the reaction occurs due to the enhanced activation [24]. Excessively high temperatures can also lead to excessive energy consumption. In this experiment, we controlled the heating temperature between 140 °C and 280 °C. Figure 4a and Figure S4a illustrate the response values of pure SnO_2_, 0.5 wt%-PdPt-SnO_2_, 1.0 wt%-PdPt-SnO_2_, 1.0 wt%-Pd-SnO_2_, 1.0 wt%-Pt-SnO_2_, and 1.5 wt%-PdPt-SnO_2_ to 10 ppm formaldehyde. We observed that, at 140 °C, the sensor began to respond to formaldehyde, and as the temperature increased, the response value grew, reaching its maximum at 180 °C for the PdPt-decorated SnO_2_ sensors, 200 °C for the single noble metal loaded SnO_2_ sensors, and 260 °C for the pristine SnO_2_ sensor. However, as the temperature increased further, the response value began to decrease, leading us to select 180, 200, and 260 °C as the optimal operating temperatures for these sensors. Notably, during this period, the 1.0 wt%-PdPt-SnO_2_ exhibited the highest response to 10 ppm formaldehyde compared to the other three materials, with a response value of *R*_a_/*R*_g_ = 8.2 at 180 °C. In contrast, the pristine SnO_2_ sensor only reached its optimal working temperature at 260 °C, which is 60 °C higher than the Pd- or Pt-decorated SnO_2_ sensors and 80 °C higher than the three PdPt-decorated SnO_2_ sensors. The modification effectively reduces the potential barrier while enhancing the surface activity of the material [25,26].

After determining the optimal working temperature, we assessed the selectivity of these gas-sensing materials under the given conditions. The sensors were tested with six gases: 1000 ppm CH_4_, 10 ppm CO, NH_3_, NO, H_2_S, and HCHO (Figure 4b and Figure S4b). All four sensors exhibited the highest response values to HCHO, a trend that is further illustrated in the radar chart (Figure 4c and Figure S4c). To quantify selectivity, the selectivity coefficient between HCHO and H_2_S was calculated. The response ratios were as follows: 1.6 for SnO_2_; 2.1 for Pd-SnO_2_ or Pt-SnO_2_; and 2.6, 2.8, and 2.6 for different PdPt-SnO_2_-based sensors. These results confirm that the PdPt-modified sensors demonstrate improved selectivity toward HCHO compared to the other gases.

**Figure 5:**
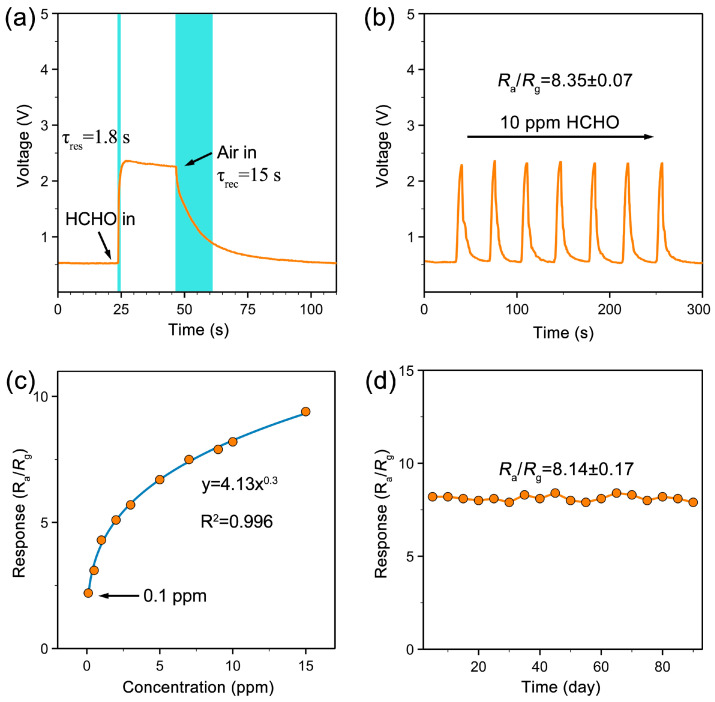
(**a**) Response curve and (**b**) cyclic tests for 1.0 wt%-PdPt-SnO_2_-based sensor to 10 ppm HCHO. (**c**) Relationship fitting between response value and HCHO concentration (0.1~15 ppm). (**d**) Long-term stability tests for 1.0 wt%-PdPt-SnO_2_-based sensor to 10 ppm HCHO. The working temperature was 180 °C, and relative humidity was 50%.

After evaluating the four materials, 1.0 wt%-PdPt-SnO_2_ demonstrated the best overall performance, prompting us to conduct further testing on it. The sensor’s response and recovery times to 10 ppm HCHO at 180 °C were 1.8 s and 15 s, respectively (Figure 5a). The short response time is crucial for warning systems, and 1.8 s is particularly rapid for practical applications. Continuous testing revealed that the 1.0 wt%-PdPt-SnO_2_ sensor maintained excellent signal reliability and stability, with a response value of *R*_a_/*R*_g_ = 8.35 ± 0.07 over seven cycles (Figure 5b). Establishing a clear correlation between gas concentration and sensor response is essential for accurately converting electrical signals into gas concentrations [27]. By fitting the response values (y) obtained from testing the 1.0 wt%-PdPt-SnO_2_ sensor at different formaldehyde concentrations (x), we derived the relationship y = 4.13x^0.3^ with a goodness of fit R^2^ = 0.996, indicating a strong correlation (Figure 5c). The sensor coating typically experiences a gradual decline in performance during long-term use. In this work, nanoparticles synthesized using a non-template, non-surfactant method retained their natural state, contributing to excellent long-term thermal stability. After the aging process, the response curve remained stable over 90 days with an average response value of *R*_a_/*R*_g_ = 8.14 ± 0.17 (Figure 5d). This stability highlights the potential for reliable long-term use in sensing applications.

Humidity interference is a common challenge in gas-sensor detection. To address this, we tested the six types of sensors under varying relative humidity conditions (Figure S5). When the relative humidity reached 60%, a slight decrease in sensor performance was observed. However, even in a 90% humidity atmosphere, the sensors still exhibited clear responses, demonstrating their strong anti-interference ability.

### 3.3. Mechanism Analysis

As is well known, the gas-sensing mechanism of metal oxide semiconductors (MOS) is primarily governed by surface processes, where the surface state and oxygen adsorption capacity play crucial roles. The sensing mechanism involves the redox reactions between the gases and the adsorbed oxygen species (*O*_2_^−^, *O*^−^, and *O*^2−^). When the sensing material is exposed to air, oxygen molecules adsorb onto its surface, causing electrons to transfer from the material to the oxygen molecules, leading to the formation of various oxygen ions. This results in an increase in the material’s resistance. The primary chemical equations involved are as follows:

(1) $$O_{2}\left(gas\right)\to O_{2}\left(ads\right)\;$$

(2) $$O_{2}\left(ads\right)+e^{-}\to O_{2}^{-}\left(ads\right)\;$$

(3) $$O_{2}^{-}(ads)+e^{-}\to 2O^{-}\left(ads\right)$$

(4) $$O^{-}(ads)+e^{-}\to O^{2-}\left(ads\right)$$

At the operating temperature of the sensing material (180 °C in this study), formaldehyde undergoes a redox reaction with the adsorbed oxygen ions. During this process, the oxygen ions release the captured electrons back into the conduction band of the MOS material, SnO_2_. As a result, the carrier concentration increases, leading to a decrease in the material’s resistance [28], shown as Equations (5) and (6):

(5) $$HCHO\left(gas\right)\to HCHO\left(ads\right)$$

(6) $$HCHO\left(gas\right)+2O^{2-}\to {CO}_{2}+H_{2}O+{4e}^{-}\;$$

**Figure 6:**
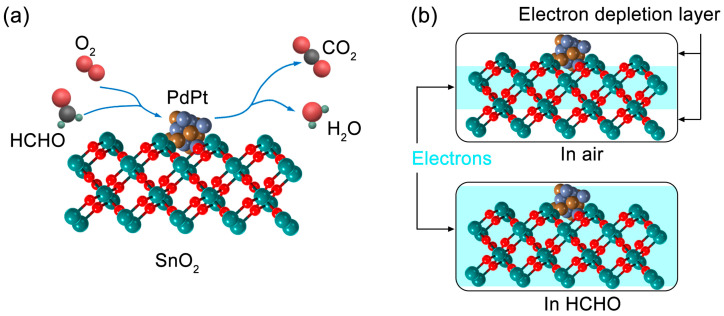
Illustration of (**a**) the reaction mechanism between PdPt-SnO_2_ and HCHO molecules and (**b**) electron transmission model of PdPt-SnO_2_ in air and HCHO atmosphere.

As shown in Figure 6, O_2_ is adsorbed onto the surface of PdPt-SnO_2_, where it reacts with formaldehyde (HCHO) to produce CO_2_ and H_2_O. During this reaction, the electron depletion layer in SnO_2_ widens due to the adsorption of O_2_. When HCHO reacts with O_2_, electrons are transferred back to SnO_2_, causing the electron depletion layer to become thinner. This change in the electron depletion layer alters the conductivity of SnO_2_, which is detected by the external circuit, generating gas-sensitive sensing signals [29]. The PdPt bimetallic component acts as a catalyst in this reaction, effectively lowering the activation energy and accelerating the reaction rate [30], and the interfaces between SnO_2_ and PdPt nanoparticles may undertake the main reaction function [31].

### 3.4. Practical Application

**Figure 7:**
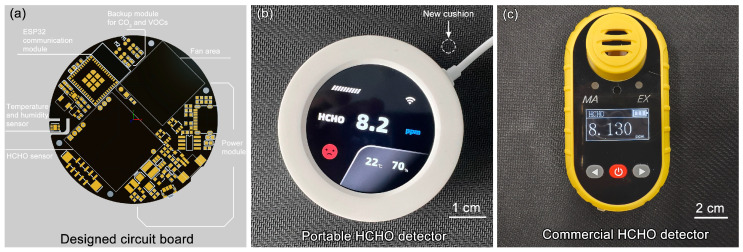
(**a**) Designed circuit board and (**b**) portable HCHO detector; (**c**) commercial HCHO detector on new cushion.

The goal of scientific research is to translate laboratory findings into practical applications [32]. After thorough validation of the material properties, a 1.0 wt%-PdPt-SnO_2_-based sensor was chosen to develop a portable HCHO detector. A circuit board integrating communication, power, temperature and humidity control, fan control, a CO_2_ and VOCs backup module, and the HCHO sensor module was designed (Figure 7a). The circuit board was then assembled into the HCHO detector, which was placed on a new plastic cushion for detection. The screen displayed an HCHO concentration of 8.2 ppm (Figure 7b), which is very close to the value 8.130 ppm collected by a commercial HCHO gas detector (Figure 7c, purchased from Shenzhen Pusheng Sensing Technology Co., Ltd., Shenzhen, China), indicating that some treatment measures are necessary before safe usage. This result demonstrates the sensor’s effectiveness in real-world applications and highlights the importance of ensuring air quality before use.

## 4. Conclusions

In conclusion, we utilized a straightforward synthesis approach to effectively load PdPt noble metals onto the surface of SnO_2_ nanoparticles. Notably, this process was accomplished without the use of surfactants or templates. The successful loading significantly enhanced the gas-sensing properties towards HCHO, with a particular emphasis on stability, a factor of utmost importance for industrial applications. Furthermore, we developed a portable detector designed to monitor HCHO emissions from new furniture. This research not only bridges the chasm between laboratory-based research and real-world application but also propels scientific advancement. It vividly demonstrates the promising potential for industrial utilization, presenting a practical solution that combines scientific innovation with practical utility in the field of gas-sensing technology.

Looking ahead, we will explore the application of this series of gas-sensing materials in detecting other harmful gases in addition to HCHO, such as VOCs (volatile organic compounds), to expand the scope of detection. Moreover, we plan to collaborate with relevant industries to conduct large-scale field tests of the portable detector, collect more real-world data, and continuously improve the product according to the feedback. In this way, we hope to make this technology more mature and widely used in various industrial and daily-life scenarios.

## Figures and Tables

**Table 1:** The Pd and Pt element ICP-OES information of the decorated SnO_2_.

Materials	Average Amount (Pd wt%)	Average Amount (Pt wt%)	Standard Error (wt%)
0.5 wt%-PdPt-SnO_2_	0.27	0.24	0.01
1.0 wt%-Pd-SnO_2_	0.98	-	0.02
1.0 wt%-Pt-SnO_2_	-	1.00	0.02
1.0 wt%-PdPt-SnO_2_	0.51	0.48	0.01
1.5 wt%-PdPt-SnO_2_	0.77	0.75	0.02

## Data Availability

The authors confirm that the data supporting the findings of this study are available within the article.

## Supplementary Materials

The following supporting information can be downloaded at: https://www.mdpi.com/article/10.3390/s25051627/s1, Figure S1. The XRD patterns of Pd-SnO_2_ and Pt-SnO_2_. Figure S2. XPS survey spectrum of 1.0 wt%-PdPt-SnO_2_. Figure S3. SEM image of MEMS sensor with SnO_2_ coating on the surface. Figure S4. (a) Responses at various working temperatures; (b) selectivity tests to 10 ppm of different gases; and (c) transformed radar charts for the sensors based on Pd-SnO_2_, Pt-SnO_2_, and PdPt-SnO_2_ nanoparticles in response to 10 ppm HCHO. Figure S5. Humidity interference tests for the MEMS sensors.

## Abbreviations

The following abbreviations are used in this manuscript:

MOS	Metal oxide semiconductor
MEMS	Micro-electro-mechanical system
XRD	X-ray diffraction pattern
TEM	Transmission electron microscope
HRTEM	High-resolution TEM
EDX	Energy dispersive X-ray spectroscopy
FESEM	Field emission scanning electron microscope
ICP-OES	Inductively coupled plasma optical emission spectrometer
XPS	X-ray photoelectron spectra
