# Supplementary material for: Industrial Potential of Formaldehyde Gas Sensor Based on PdPt Bimetallic Loaded SnO2 Nanoparticles
Source: Sensors (Basel). 2025 Mar 6;25(5):1627. doi: 10.3390/s25051627 (PMC11902779; doi:10.3390/s25051627)
Supplement: Supplementary file 1 [file sensors-25-01627-s001.zip › sensors-3493141-supplementary.pdf]

Supporting figures

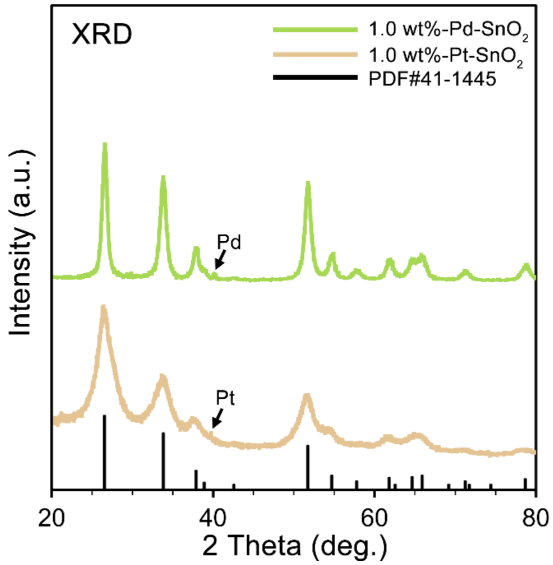

Figure S1. The XRD patterns of Pd-SnO_2 and Pt-SnO_2.

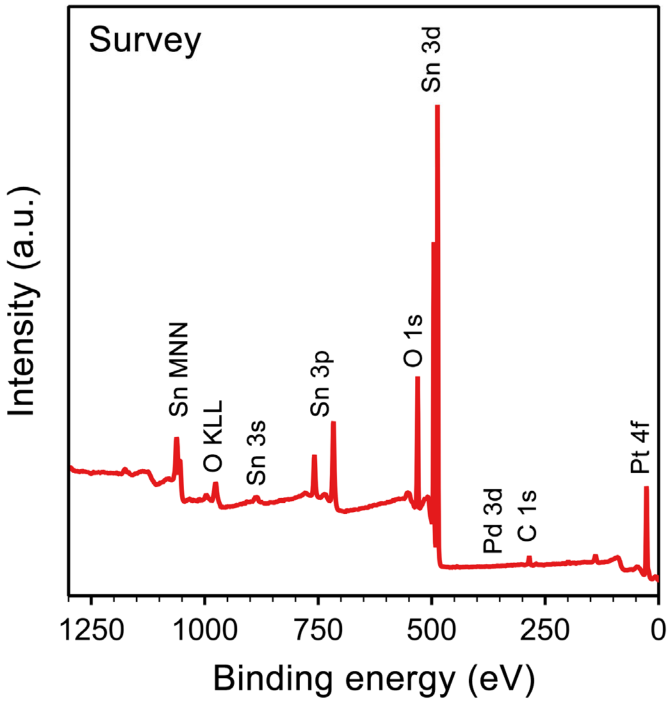

Figure S2. XPS survey spectrum of 1.0 wt%-PdPt-SnO_2.

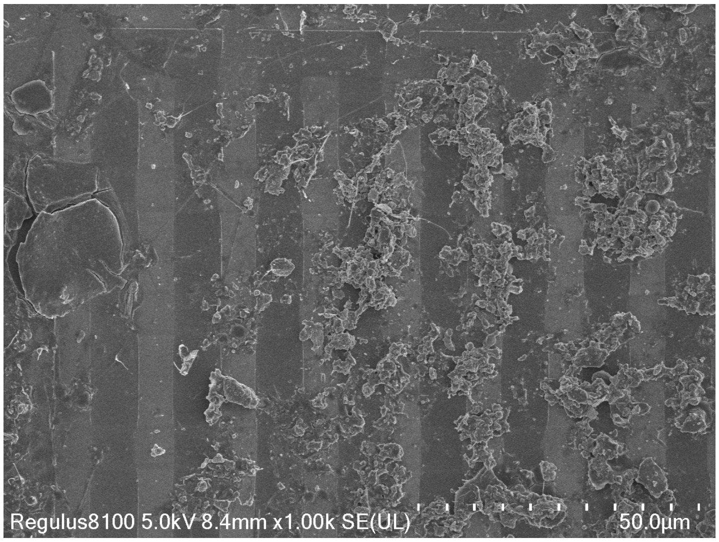

Figure S3. SEM image of MEMS sensor with SnO_2 coating on the surface.

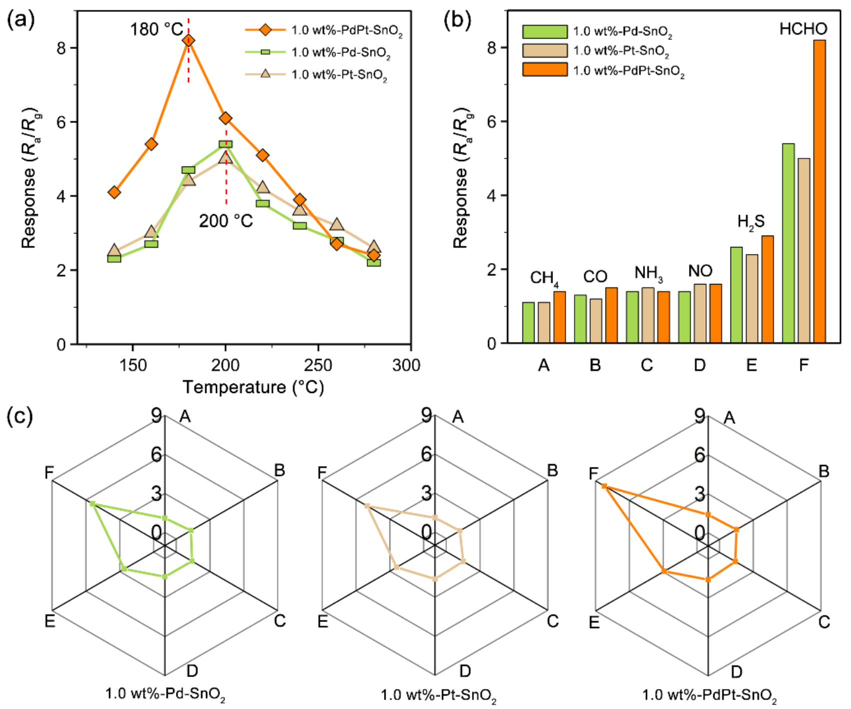

Figure S4. (a) Responses at various working temperatures; (b) selectivity tests to 10 ppm of different gases; and (c) transformed radar charts for the sensors based on Pd-SnO_2, Pt-SnO_2, and PdPt-SnO_2 nanoparticles in response to 10 ppm HCHO.

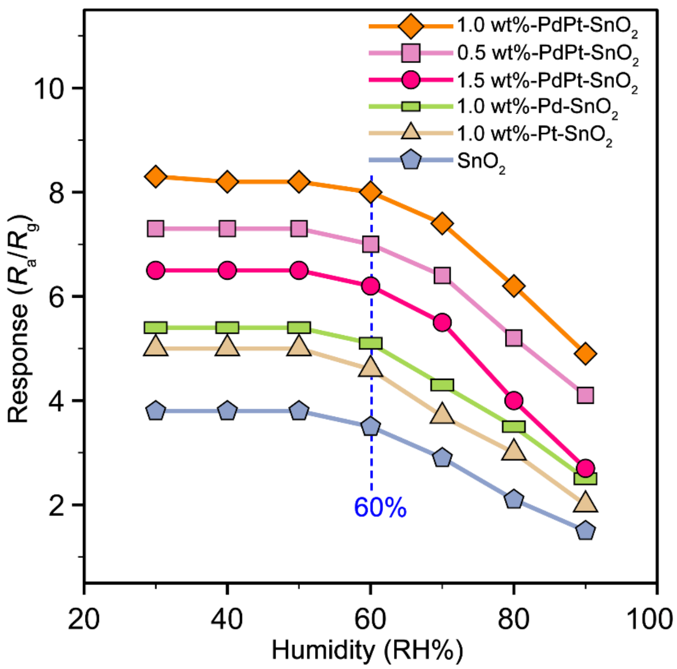

Figure S5. Humidity interference tests for the MEMS sensors.
